# Enterobactin induces the chemokine, interleukin-8, from intestinal epithelia by chelating intracellular iron

**DOI:** 10.1080/19490976.2020.1841548

**Published:** 2020-11-10

**Authors:** Piu Saha, Beng San Yeoh, Xia Xiao, Rachel M. Golonka, Ahmed A. Abokor, Camilla F. Wenceslau, Yatrik M. Shah, Bina Joe, Matam Vijay-Kumar

**Affiliations:** a UT Microbiome Consortium, Department of Physiology & Pharmacology, University of Toledo College of Medicine and Life Sciences, Toledo, OH, USA; b Center for Systems Biology, Massachusetts General Hospital, Harvard Medical School, Boston, MA, USA; c Department of Molecular and Integrative Physiology, University of Michigan Medical School, Ann Arbor, MI, USA; d Division of Gastroenterology, Department of Internal Medicine, University of Michigan Medical School, Ann Arbor, MI, USA; e Rogel Cancer Center, University of Michigan Medical School, Ann Arbor, MI, USA

**Keywords:** Siderophores, enterochelin, IL-8, labile iron pool, lipocalin 2

## Abstract

Iron is an indispensable nutrient for both mammals and microbes. Bacteria synthesize siderophores to sequester host iron, whereas lipocalin 2 (Lcn2) is the host defense protein that prevent this iron thievery. Enterobactin (Ent) is a catecholate-type siderophore that has one of the strongest known affinities for iron. Intestinal epithelial cells (IECs) are adjacent to large microbial population and are in contact with microbial products, including Ent. We undertook this study to investigate whether a single stimulus of Ent could affect IEC functions. Using three human IEC cell-lines with differential basal levels of Lcn2 (*i.e*. HT29 < DLD-1 < Caco-2/BBe), we demonstrated that iron-free Ent could induce a dose-dependent secretion of the pro-inflammatory chemokine, interleukin 8 (IL-8), in HT29 and DLD-1 IECs, but not in Caco-2/BBe. Ent-induced IL-8 secretion was dependent on chelation of the labile iron pool and on the levels of intracellular Lcn2. Accordingly, IL-8 secretion by Ent-treated HT29 cells could be substantially inhibited by either saturating Ent with iron or by adding exogenous Lcn2 to the cells. IL-8 production by Ent could be further potentiated when co-stimulated with other microbial products (i.e. flagellin, lipopolysaccharide). Water-soluble microbial siderophores did not induce IL-8 production, which signifies that IECs are specifically responding to the lipid-soluble Ent. Intriguingly, formyl peptide receptor (FPR) antagonists (i.e. Boc2, cyclosporine H) abrogated Ent-induced IL-8, implicating that such IEC response could be, in part, dependent on FPR. Taken together, these results demonstrate that IECs sense Ent as a danger signal, where its recognition results in IL-8 secretion.

## Graphical abstract

**Figure:**
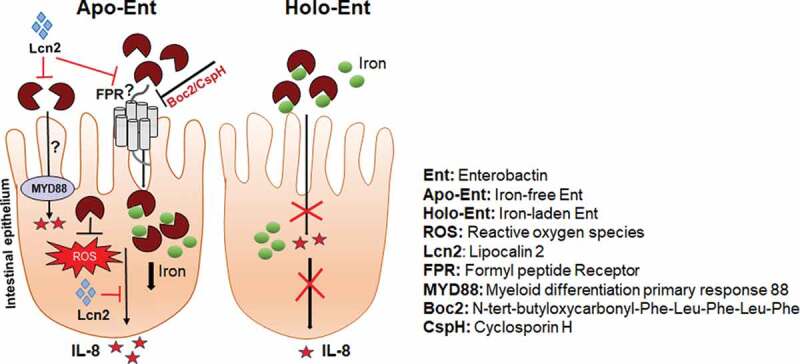

## Introduction

The apical surface of the gastrointestinal tract is lined with a single layer of intestinal epithelial cells (IECs) that provide a physical barrier to demarcate the mucosa from the luminal bacteria collectively denoted as the gut microbiota.^1^ IECs initiate a cross-talk with the innate immune system when they sense intestinal microorganisms, gut metabolites, and bacteria-derived stimulators. This includes activating pattern recognition receptors, secreting humoral factors and chemokines, and upregulating the expression of mucosal proteins that are essential for securing gut barrier function.^2^ To highlight one example, the Gram-negative bacterial component flagellin can strongly induce the secretion of the chemoattractant interleukin (IL)-8 (*alias* human CXCL8) from IECs,^3,4^ which is essential for recruiting immune cells, like neutrophils, to the site of microbial insult. The responsibility of IECs to be accessory immune cells is critical for maintaining gut homeostasis because defects in intestinal epithelial barrier integrity would allow for passage of harmful luminal contents that contribute to the development of inflammation-associated diseases, including inflammatory bowel disease and metabolic endotoxemia.^5-7^

In addition to recognizing danger-associated molecular patterns from gut microbes, IECs serve as nutrient sensors that can regulate bactericidal responses to protect against enteric pathogens during nutrient fluctuations.^8^ Generally, the gut microbiota and host are in a constant ‘*tug-of-war*’ for vital nutrients, where both sides have developed adaptive mechanisms in attempt to gain the upper hand. Iron is one such indispensable nutrient to support bacterial growth and to maintain a wide range of host physiological functions, including oxygen transport, electron transport, and DNA synthesis. To actively limit the bioavailability of iron, the host expresses high levels of circulating iron-binding proteins (i.e. transferrin). Moreover, the host can also potentiate a state of systemic iron deficiency (*alias* hypoferremia of inflammation) during infection or inflammation.^9^ To gain access to the host iron pool, various commensal and pathogenic bacteria secrete iron chelators, called siderophores, to wrestle this micronutrient from host iron-binding proteins.^10^ One such siderophore, enterobactin (Ent; *alias* enterochelin, an amino acid derivative of 2,3 dihydroxy N-benzoylserine lactone), is renowned as the archetypal, catecholate-type siderophore expressed by Gram-negative bacteria with the highest known affinity toward the ferric (Fe^+3^) form of iron.^11,12^ Its iron chelation potency in combination with its high membrane affinity and hydrophobicity^13^ makes Ent capable of penetrating cellular membranes and depleting intracellular iron storages in mammalian cells.^14-16^

Besides functioning as an iron chelator, Ent also benefits their producers through other non-canonical functions such as facilitating bacterial colonization,^17^ quorum sensing and biofilm formation,^18^ and mitigating oxidative stress.^19-21^ In addition, we have shown that Ent can impede neutrophil functions by inhibiting myeloperoxidase^22,23^ and the formation of neutrophil extracellular traps.^16^ To thwart the negative effects of Ent, the host synthesizes the innate immune protein lipocalin 2 (Lcn2; *alias* neutrophil gelatinase-associated lipocalin [NGAL], siderocalin, or 24p3) to sequester both iron-bound and iron-free Ent.^24^ Such maneuver has been shown to be a potent host defense against intestinal^25^ and lung^26^ infections by Ent-producing *Enterobacteriaceae*. Previous studies have also shown that lung epithelia secrete interleukin 8 (IL-8) in response to Ent alone or in combination with Lcn2.^27,28^ However, it remains unclear as to whether such a response could be recapitulated in IECs, which are more likely to be exposed to Ent-producing gut commensals under normal physiological conditions and in the absence of infection.

Herein, we investigated whether Ent could trigger an induction of IL-8 in human colonic IECs. Collectively, our study indicates that a single stimulus of iron-free, but not iron-bound, Ent could induce IL-8 secretion from IECs, where this was dependent on its ability to chelate the intracellular labile iron pool (LIP). Using three human IEC cell-lines that express differential basal levels of Lcn2 (*i.e*. HT29 < DLD-1 < Caco-2/BBe), we demonstrate that Ent-induced IL-8 and intracellular iron chelation were more pronounced in cells expressing lower levels of Lcn2. Inhibiting formyl peptide receptor (FPR) with an antagonist (i.e. Boc2, cyclosporine H) mitigated IL-8 secretion in Ent-treated IECs, indicating a possible role of FPR in mediating the IL-8 response to Ent.

## Results

### Ent chelates intracellular iron in human model of intestinal epithelia

**Figure 1.**
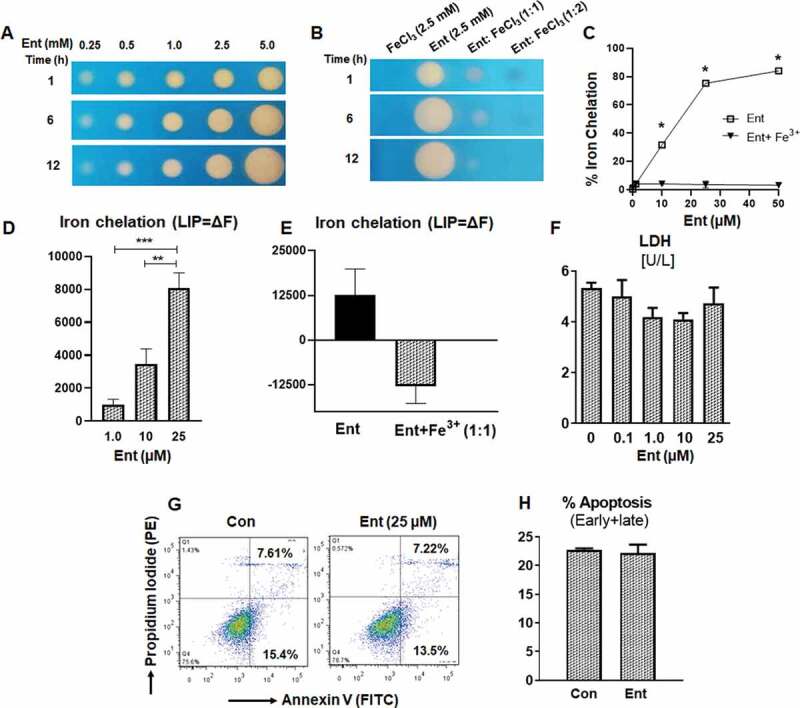
Enterobactin, chelates intracellular iron in human intestinal epithelia. (a) Iron chelation as indicated by the formation of an orange halo by Ent (0.25 mM to 5 mM) on CAS agar plate over different time periods (1, 6 and 12 h). (b) Iron chelation of FeCl_3_ (2.5 mM), Ent (2.5 mM), Ent: FeCl_3_ (1:1 ratio) and Ent: FeCl_3_ (1:2 ratio) on CAS agar plate over different time periods (1, 6 and 12 h). (c) Line graphs indicate the relative iron chelation activity of Ent (0–50 µM) and Ent: FeCl_3_ (1:1 ratio) detected via CAS liquid assay. (d-e) HT29 cells were incubated with 0.5 μM calcein-AM for 15 min and then treated with iron-free or iron-bound Ent (0–25 µM) for 3 h in serum-free media supplemented with 1% penicillin-streptomycin. After washing, iron chelation (LIP = ΔF) was quantitated by ﬂow cytometry. (d) Bar graph represents the iron chelation in HT29 cells after 3 h of iron-free Ent (0–25 µM) treatment. (e) Bar graph shows the iron chelation in HT29 cells after 3 h of Ent (25 µM) and Ent+ Fe^3+^ (1:1 ratio) treatment. (f) HT29 cells (2.0 x10^6^ cells/ml) were treated with Ent (0–25 µM) for 24 h in serum-free media supplemented with 1% penicillin-streptomycin. The release of lactate dehydrogenase (LDH) in the culture supernatant was measured. (g-h) Ent (25 µM)-treated HT29 cells (24 h) were analyzed for cellular apoptosis measured by flow cytometry using Annexin-V/Propidium Iodide positivity. (g) Representative dot plots show the percentage of early and late apoptosis in control and Ent treated HT29 cells. (h) Bar graph indicates the % apoptosis (% Annexin-V+ Propidium Iodide, both early and late apoptosis) at 24 h treatment. Con denotes cells treated with DMSO as vehicle control. *In vitro* assays were performed in triplicates and data represented as mean ± SEM. * *p* < .05, ** *p* < .01, and *** *p* < .001

Iron-limiting conditions prompt bacteria to synthesize siderophores, which are low molecular weight (500–1,500 Da), chemical iron chelators that possess a higher affinity toward iron compared to host iron-binding proteins.^29^ One such siderophore, enterobactin (Ent), is a triscatechol derivative of a cyclic triserine lactone expressed by diverse Gram-negative bacteria. When assayed for iron-binding function on a chrome azurol S (CAS) plate, Ent exhibited a dose- and time-dependent formation of an orange colored halo that represents iron chelation (Figure 1(a)). Ent pre-saturated with FeCl_3_ at a 1:1 ratio drastically diminished the halo appearance on CAS plate, indicating that Ent binding to an equimolar concentration of iron is sufficient to prevent further iron chelation (Figure 1(b)). To quantify this observation, we performed a more sensitive CAS liquid assay^30^ with Ent in the presence or absence of equimolar FeCl_3_. As expected, iron-bound Ent did not display any iron-chelating activity (Figure 1(c)). Comparatively, the iron-chelating activity of iron-free Ent increased in a dose-dependent fashion, where 10, 25 and 50 µM Ent achieved around 30%, 75% and 84% chelation, respectively (Figure 1(c)).

Next, we sought to determine whether iron-free Ent could chelate the intracellular iron pool (LIP) in the human intestinal epithelial cell line HT29.^31^ By using the calcein-AM method,^32,33^ we noted that iron-free Ent could chelate the intracellular LIP from HT29 cells in a dose-dependent manner (Figure 1(d)). As anticipated, iron-laden Ent failed to chelate intracellular iron from HT29 cells (Figure 1(e)). To determine whether iron chelation by Ent could affect cell viability because iron supports vital functions, including cellular respiration, we assayed the cell-free culture supernatant for lactate dehydrogenase (LDH), whose release indicates cytosolic leakage and can be also used as an index for cell death.^34^ However, we did not observe any cytotoxic effects of Ent on HT29 cells (Figure 1(f)). To affirm this result, we stained Ent-treated HT29 cells with annexin-V and propidium iodide, whose co-positivity would indicate that these cells have undergone late apoptosis. Consistent with our previous analysis, we did not detect any apoptotic effects of Ent on HT29 cells (Figure 1(g,h)).

### Iron-free, but not iron-bound, Ent induces secretion of the chemokine, IL-8 in IECs

**Figure 2.**
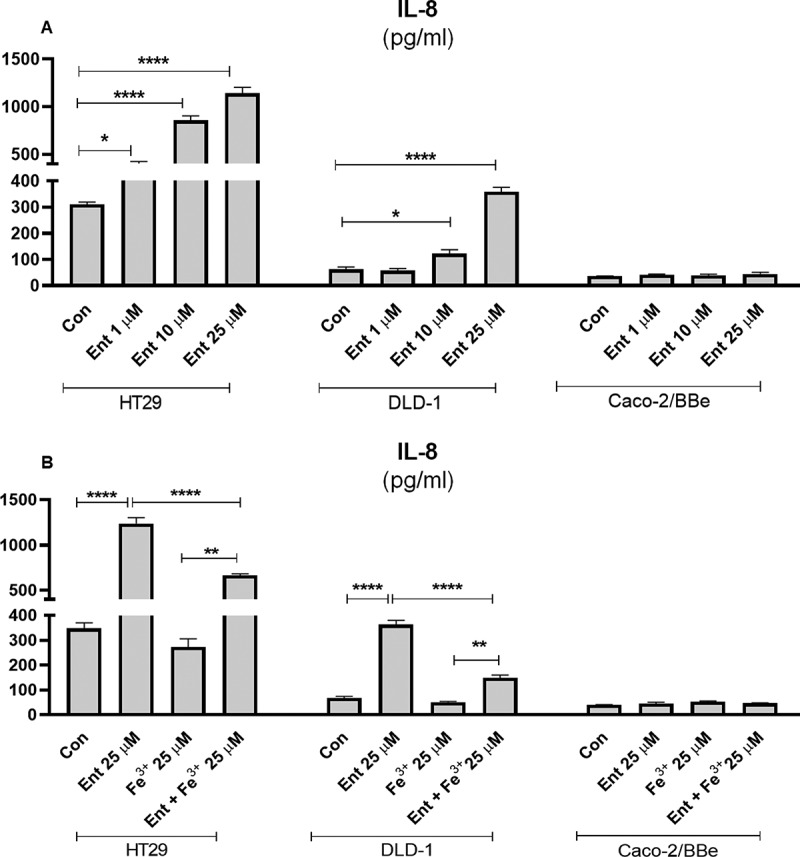
Pro-inflammatory effects of Ent on models of human intestinal epithelia. (a) HT29, DLD-1 and Caco-2/BBe cell monolayers were incubated with Ent (0–25 µM) for 24 h in serum-free media supplemented with 1% penicillin-streptomycin and culture supernatants were collected and assessed for IL-8 secretion by ELISA. (b) The monolayers of HT29, DLD-1, and Caco-2/BBe cells were incubated overnight with Ent (25 µM), FeCl_3_ (Fe^3+^) or Ent with an equimolar concentration of FeCl_3_ and supernatants were analyzed for IL-8. Con denotes cells treated with DMSO as vehicle control. *In vitro* assays were performed in triplicates and data represented as mean ± SEM. * *p* < .05, **p < .01, *** *p* < .001 and **** *p* < .0001

Intestinal epithelial cells (IECs) are the border defense for segregating our body from the gut bacteria that corral the intestinal lumen. As such, this cell-type would be continuously exposed to diverse microbial products, including siderophores like Ent that can reach a high concentration in the mucus layer.^17^ To assess whether Ent could induce the secretion of the chemokine, IL-8, from IECs, we added varying concentrations of Ent to monolayer cultures of model intestinal epithelia (i.e. HT29, DLD-1 and Caco-2/BBe). We observed a dose-dependent increase of IL-8 secretion from HT29 and DLD-1 cells after 24 h Ent treatment, with the former exhibiting a more prominent response than the latter (Figure 2(a)). Of note, Ent at a concentration of 1 µM was sufficient to induce a > 2-fold increase in IL-8 secretion from HT29 cells (Figure 2(a)). Comparatively, Caco-2/BBe cells were relatively refractory to Ent-induced IL-8 (Figure 2(a)).

To test whether the induction of IL-8 from IECs requires Ent to be in its iron-free state, we challenged the IEC cell-lines with Ent pre-saturated with an equimolar ratio of ferric iron. Indeed, both HT29 and DLD-1 cells secreted two-fold less IL-8 in response to iron-laden Ent relative to their respective counterparts that were treated with iron-free Ent (Figure 2(b)). No differences were observed in Caco-2/BBe cells (Figure 2(b)). Of note, 25 µM of ferric iron (Fe^3+^) alone did not elevate IL-8 from any of the cell-lines. As HT29 cells displayed the strongest response to Ent, we focused most of our subsequent experiments on this IEC cell-line.

**Figure 3.**
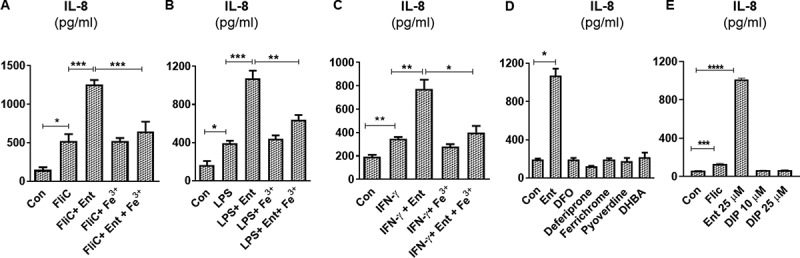
Ent triggers inflammatory signals in human intestinal epithelia in the presence of IL-8 inducers. HT29 monolayers were pre-incubated with either (a) FliC (100 ng/ml, 1 h), (b) LPS (1 µg/ml, 1 h) or (c) IFN-γ (100 IU, 1 h) and treated with Ent (25 µM), FeCl_3_ (Fe^3+^) or Ent with an equimolar concentration of FeCl_3_ for 24 h in serum-free media supplemented with 1% penicillin-streptomycin and supernatants were assayed for IL-8 via ELISA. DMSO served as control. (d) HT29 cells were stimulated overnight with 25 µM of either Ent, DFO, deferiprone, ferrichrome, pyoverdine or DHBA in serum-free media supplemented with 1% penicillin-streptomycin and assayed for IL-8. (e) HT29 cells were stimulated overnight with either Ent (25 µM), FliC (100 ng/ml) or 2,2 dipyridyl (DIP, 0–25 µM) in serum-free media supplemented with 1% penicillin-streptomycin. After stimulation, culture medium was collected, assayed for IL-8. Con denotes cells treated with DMSO as vehicle control. *In vitro* assays were performed in triplicates and data represented as mean ± SEM. * *p* < .05, **p < .01, *** *p* < .001, and **** *p* < .0001

Lipopolysaccharide (LPS) and flagellin (FliC) are two abundant microbial components that are known to induce an inflammatory response through activation of toll-like receptor (TLR) 4 and TLR5, respectively. We asked whether Ent-induced IL-8 secretion could be affected in the presence of either microbial ligands. When we added Ent to HT29 cells that had been primed with FliC, we observed an additive effect in IL-8 secretion when compared to cells treated with FliC alone (Figure 3(a)). Treatment with either Fe^3+^ or vehicle control did not alter IL-8 secretion in FliC-primed HT29 cells (Figure 3(a)). Yet, consistent with our prior observation with iron-laden Ent, the addition of Ent+Fe^3+^ did not further increase the IL-8 upregulated by FliC (Figure 3(a)). Similar outcomes were observed when the experiment was repeated with LPS-primed HT29 cells receiving either iron-free or iron-laden Ent (Figure 3(b)). Next, we asked whether pre-treating HT29 with interferon gamma (IFN-γ), a type II interferon known to upregulate IL-8,^35^ could interfere with Ent-induced IL-8 secretion. This was not the case as iron-free Ent, but not iron-laden Ent, was still capable of inducing IL-8 secretion in HT29 cells pre-treated with IFN-γ (Figure 3(c)). Collectively, the additive effect in IL-8 secretion observed in these experiments indicates that IECs remain responsive to Ent challenge despite already being in an inflammatory state following stimulation with either FliC, LPS or IFN-γ. We could infer that Ent may upregulate IL-8 in a noncompetitive manner or independently of FliC, LPS or IFN-γ, though further studies are needed to discern the extent to which their downstream pro-inflammatory signaling pathways overlap.

The requirement for Ent to be in its iron-free state to induce IL-8 prompted us to test whether such IEC response could be recapitulated using other bacterial or fungal iron chelators. However, we did not observe any induction in IL-8 secretion in HT29 cells stimulated with 25 µM of either deferrioxamine (DFO) from *Streptomyces pilosus*, ferrichrome from *Ustilago sphaerogena*, pyoverdine from *Pseudomonas fluorescens*, or 2,3 dihydroxybenzoic acid (DHBA, the precursor molecules that contribute to the formation of Ent) (Figure 3(d)). The synthetic form of DFO, namely Deferiprone, also did not induce IL-8 secretion from HT29 cells (Figure 3(d)). The discrepancy between Ent and the various siderophores assayed herein could be influenced by the lower lipid solubility in the latter, which could reduce their cell membrane permeability. To address this notion, we next tested whether 2,2 dipyridyl (DIP), a highly cell-permeable, hydrophilic ferrous iron chelator could induce IL-8 secretion from HT29 cells. DIP at 10 and 25 µM failed to induce IL-8 secretion from HT29 cells (Figure 3(e)), suggesting that differences in membrane permeability alone is not sufficient to explain IL-8 induction or lack thereof from IECs by siderophores. We consider that such IL-8 induction may be driven by the degree of iron chelation of Ent, DFO, and DIP whose K_a_ values are 10^49^, 10^31^, 10^28^ M^−1^, respectively.^36-40^ It is reported that DFO, at a high concentration of 0.2 mM, can trigger the production of IL-8 in human IECs by activating the ERK1/2 and p38 kinase pathways.^41,42^

### Ent impedes reactive oxygen species generation and Lcn2 secretion in IECs

**Figure 4.**
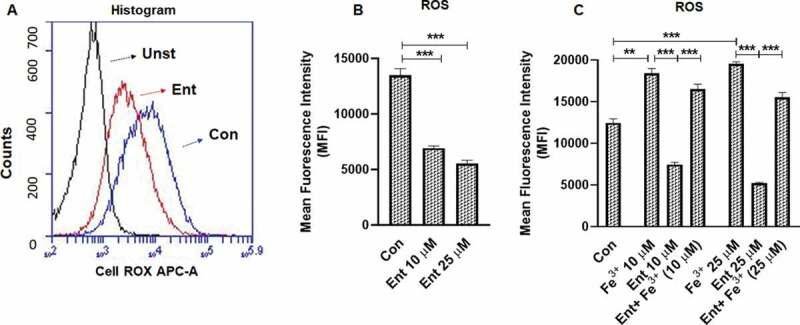
Ent inhibits reactive oxygen species (ROS) generation in intestinal epithelia. HT29 cells were incubated with Ent (0–25 µM), Fe^3+^ (0–25 µM) or Ent+ Fe^3+^ (1:1 ratio) for 24 h in serum-free media supplemented with 1% penicillin-streptomycin and ROS generation was measured using CellROX® Deep Red dye. (a) Histograms represent the flow cytometric analysis of intracellular ROS generation in basal and Ent treated intestinal epithelial cells quantified as MFI. (b) Bar graphs represented the dose dependent effects of Ent on basal ROS generation. (c) Bar graphs denote the effects of Ent, Fe^3^ or Ent+ Fe^3+^ (1:1 ratio) on basal ROS generation. MFI = Mean fluorescence intensity. Unst = unstained control cells treated with DMSO, Con = control cells treated with DMSO stained with CellROX® Deep Red dye. *In vitro* assays were performed in triplicates and data represented as mean ± SEM. **p < .01 and *** *p* < .001

Unlike the reactive oxygen species (ROS) generated by phagocytes to kill bacteria, IECs production of intracellular ROS [via the heme enzyme, NADPH oxidase 1 (NOX1)] is more attuned toward wound restitution^43^ and cell proliferation.^44^ Notwithstanding, reactive (Fe^+2^) free iron can catalyze the production of ROS via the Fenton reaction.^45^ Therefore, we next examined whether ROS production by IECs could be affected by Ent-mediated iron sequestration. Indeed, our results demonstrated that iron-free Ent reduced the production of basal ROS generation in IECs significantly (Figure 4(a–c)). However, iron-laden Ent failed to inhibit ROS generation in IECs (Figure 4(c)), suggesting that the chelation activity of Ent is likely required to attenuate ROS production.

**Figure 5.**
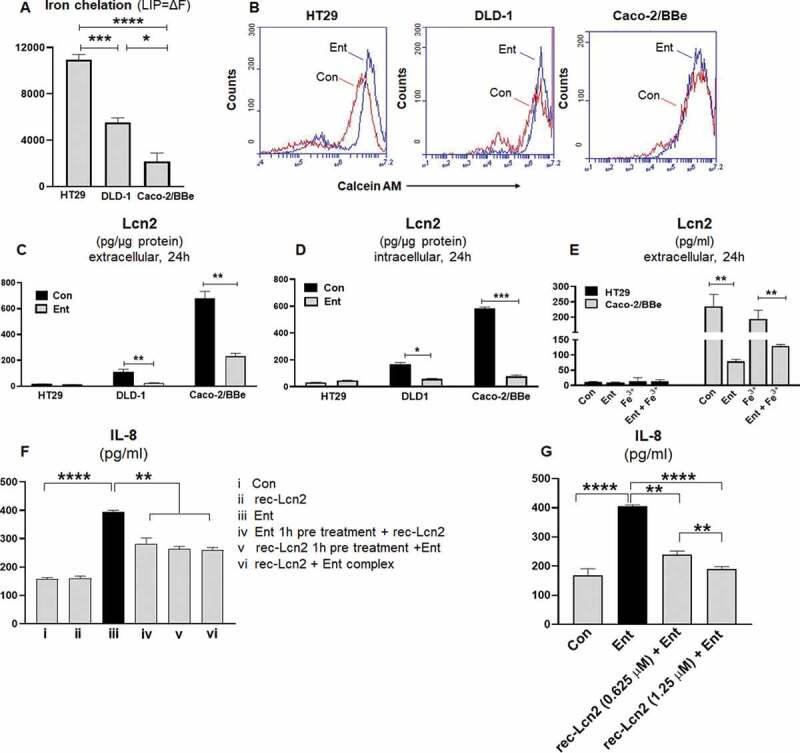
Ent treatment dampens Lcn2 secretion and host Lcn2 also impedes iron chelation property of Ent. HT29, DLD-1, and Caco-2/BBe cells were incubated with 0.5 μM calcein-AM for 15 min and then treated with iron-free or iron-bound Ent (0–25 µM) for 3 h in serum-free media supplemented with 1% penicillin-streptomycin. After washing, iron chelation (LIP = ΔF) was quantitated by ﬂow cytometry. (a) Bar graphs represent the iron chelation in HT29, DLD-1, and Caco-2/BBe cells after 3 h of iron-free Ent (0–25 µM) treatment. (b) Histograms represent the flow cytometric analysis of intracellular iron chelation in control (vehicle treatment, red) and Ent treated intestinal epithelial cells (blue). (c-d) HT29, DLD-1 and Caco-2/BBe cell monolayers were incubated with Ent (0–25 µM) for 24 h and culture supernatants were collected and assessed for (c) extracellular Lcn2 secretion and (d) intracellular Lcn2 by ELISA and normalized with the protein concentration. (e) The monolayers of HT29 and Caco-2/BBe cells were incubated overnight with Ent (25 µM), FeCl_3_ (Fe^3+^) or Ent with an equimolar concentration of FeCl_3_ and supernatants were analyzed for Lcn2. (f) HT29 monolayers were pre-incubated with either (i) Ent (25 µM, 1 h) or (ii) rec-Lcn2 (0.625 µM, 1 h) and then treated with either rec-Lcn2 or Ent or Ent+ rec-Lcn2 complex for 24 h in serum-free media supplemented with 1% penicillin-streptomycin in subsequent wells and supernatants were assayed for IL-8 via ELISA. (g) HT29 monolayers were pre-incubated with rec-Lcn2 (0.625 and 1.25 µM, 1 h) and then treated with Ent (25 µM) for 24 h in serum-free media supplemented with 1% penicillin-streptomycin in subsequent wells and supernatants were examined for IL-8 via ELISA. *In vitro* assays were performed in triplicates and data represented as mean ± SEM. * *p* < .05, **p < .01,*** *p* < .001 and **** *p* < .0001

Intracellular LIP denotes the non-ferritin-bound, redox-active iron that has been associated with cellular oxidative stress and ROS generation.^31^ Next, we asked whether the differential IL-8 response of HT29, DLD-1, and Caco2-BBe IECs could be determined by the extent to which Ent could perturb their intracellular LIP. To quantify the LIP accessible to Ent, we first stained the cells with calcein-AM, a cell-permeable dye whose fluorescence is quenched by weakly binding to intracellular LIP (30, 31) followed by adding Ent to displace LIP from calcein-AM, which resulted in a fluorescent signal. The level of cytosolic LIP accessible to Ent in HT29 and DLD-1 cells were ~5-fold and 2.5-fold higher, respectively, when compared with Caco-2/BBe cells (Figure 5(a,b)).

The degree to which Ent could chelate LIP from these cell-lines was noted to be positively correlated with their induction of IL-8 secretion in response to Ent (Figure 2). This led us to hypothesize whether factors that could sequester Ent in the extracellular milieu, such as Lcn2 secreted from IECs, may determine the degree of IL-8 response from these IECs. Accordingly, we measured the levels of Lcn2 in both cell lysate and culture supernatant from HT29, DLD-1, and Caco-2/BBe cells in presence or absence of Ent (25 µM) after 24 h treatment. Indeed, the Caco-2/BBe cells maintained significantly higher intracellular levels of Lcn2, i.e. ~33-fold and ~6-fold more compared to HT29 and DLD-1 cells, respectively (Figure 5(c,d)). Similarly, Caco-2/BBe cells also secreted more Lcn2 than HT29 and DLD-1 cells (Figure 5(c–e)). Such results suggest that the higher levels of Lcn2 secreted from Caco-2/BBe cells could counteract Ent by sequestering the latter in the extracellular compartment and thus, limiting its access to intracellular LIP. Intriguingly, despite it having minimal effect on Caco2/BBe cells in terms of LIP chelation and IL-8 induction, Ent was noted to substantially decrease the levels of both intracellular and extracellular Lcn2 in this IEC cell-line (Figure 5(c–e)). A similar reduction in Lcn2 levels in Ent-treated DLD-1 cells were also observed, but to a lesser degree (Figure 5(c–e)). These results suggest that Ent could exert effects to counteract IECs expression of Lcn2, *albeit* the underlying mechanisms as well as the broader implication of this observation requires further studies.

Next we asked whether provision of exogenous recombinant (rec) Lcn2 to HT29 cells could counteract Ent-induced IL-8 secretion. As anticipated, the addition of exogenous rec-Lcn2 one hour before treating the cells with Ent impeded their IL-8 secretion (Figure 5(f)). Similar degree of inhibition was observed when rec-Lcn2 was administered either concomitantly with Ent or one hour after Ent treatment (Figure 5(F)). A dose-dependent inhibition in Ent-induced IL-8 secretion was observed when we increased rec-Lcn2 concentration from 0.625 µM (~32% inhibition) to 1.25 µM (~50% inhibition) (Figure 5(g)). Intriguingly, in contrast to a prior report by Nelson et al.,^27^ we did not observe any difference in IL-8 secretion when the cells were treated with rec-Lcn2 alone, relative to vehicle-treated controls (Figure 5(f)). This outcome could be possibly due to that our study employed rec-Lcn2 purified from a human cell expression system, as opposed to a bacterial expression system employed by Nelson et al.^27^ It is also possible that this differing outcome could due to the cell-line or cell-type used in the present study which uses an intestinal epithelial cell line while the previous study uses respiratory epithelial cell line.^27^ Notwithstanding this discrepancy, our results suggest that Lcn2 could indeed counteract the effect of Ent on IEC cells in regards to their IL-8 response.

### Formyl peptide receptor antagonists inhibit Ent-induced IL-8 secretion in IECs

**Figure 6.**
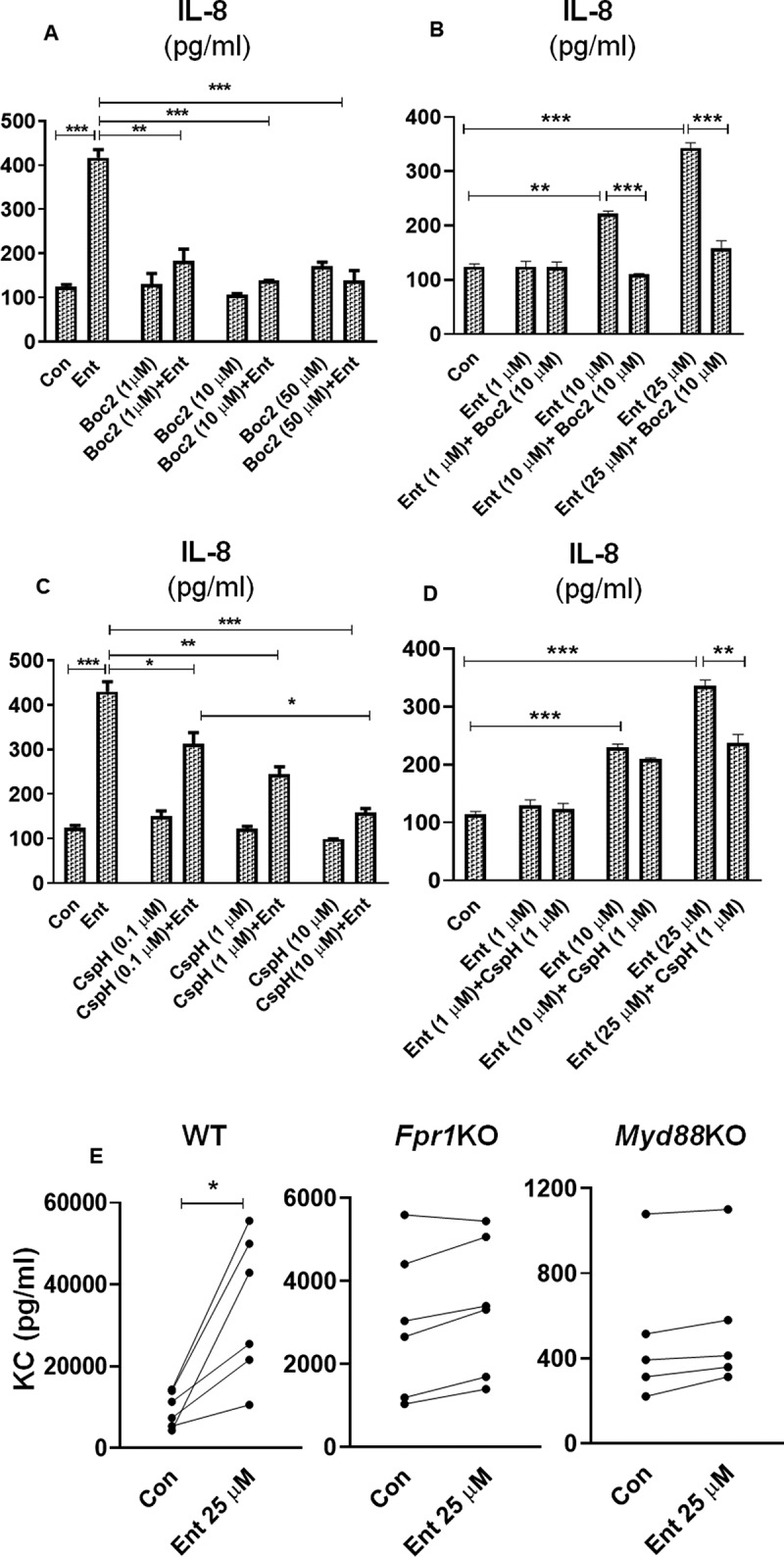
Formyl peptide receptor antagonists dampen Ent mediated IL-8 secretion in IECs. (a) HT29 cell monolayer were pre-treated with pan-FPR inhibitor Boc2 (0–50 µM, 1 h) and then stimulated with Ent (25 µM) for 24 h in serum-free media supplemented with 1% penicillin-streptomycin. Culture supernatants were analyzed for IL-8 secretion. (b) HT29 cells were pre-incubated with Boc2 (10 µM, 1 h) and then treated with Ent (0–25 µM) for 24 h. IL-8 was measured in culture supernatants. (c) HT29 cells were pre-incubated with a potent and selective FPR1 antagonist CspH (0–10 µM) for 1 h and then treated with Ent (25 µM) for 24 h in serum-free media supplemented with 1% penicillin-streptomycin. Culture supernatants were assayed for IL-8 via ELISA. (d) IECs were stimulated with CspH (1 µM) for 1 h and then treated with Ent (0–25 µM) for 24 h in serum-free media supplemented with 1% penicillin-streptomycin. Bar graphs represent the secretion of IL-8 in culture supernatant. Con denotes cells treated with DMSO as vehicle control. (e) The colon section (2 cm below the cecum) from WT, *Fpr1*KO and *Myd88*KO mice (n = 5–6, male, 8 wks old) were collected and cultured in serum-free media supplemented with 1% penicillin-streptomycin for 24 h, then stimulated with Ent (25 µM) or with DMSO as vehicle control (Con). The culture supernatants were collected and analyzed for keratinocyte-derived chemokine CXCL1 (KC). Line graphs represent the induction of KC of a sample pair (DMSO or Ent treated) from each mouse. *In vitro* assays were performed in triplicates and data represented as mean ± SEM. * *p* < .05, **p < .01 and *** *p* < .001

Formyl peptide receptors (FPRs) are expressed by various non-hematopoietic cells, including IECs, and have important roles in maintaining mucosal homeostasis and facilitating inflammatory responses.^46^ These FPRs are known to be activated via N-formyl peptides uniquely expressed by bacteria and mitochondria. We considered the possibility that Ent could interact with FPRs given that the cyclic structure of Ent contains a tri-ester lactone of 2,3-dihydroxybenzoylserine, which is formed by an amide linkage of three 2,3-dihydroxybenzoic acid groups to three L-serine units.^47^ To elucidate the role of FPRs in Ent-induced IL-8 secretion in IECs, we sought to inhibit FPR1/FPR2 signaling by using the pan-FPR antagonist, N-tert-butyloxycarbonyl-Phe-Leu-Phe-Leu-Phe (Boc2).^48^ Pre-treating HT29 cells with Boc2 at either 1, 10 or 50 µM concentrations were sufficient in preventing Ent-induced IL-8 secretion in HT29 cells (Figure 6(a,b)). The inhibitory effect of 10 µM Boc2 was not averted despite increasing the concentration of Ent from 1 to 50 µM (Figure 6(b)). To differentiate whether FPR1 or FPR2 could be the potential receptor for Ent, we next used cyclosporin H (CspH), a potent and selective competitive antagonist for the FPR1 isoform. Pre-treating HT29 cells with CspH dose-dependently inhibited Ent-induced IL-8 secretion (Figure 6(c)). The inhibitory effects of CspH, however, could be rescued by increasing the concentration of Ent, suggesting that both compounds may be competing for FPR1 binding (Figure 6(d)).

To test the requirement of FPR1 in Ent-mediated IL-8 production in IECs, we next performed an *ex vivo* culture of colon sections from wild-type (WT) and FPR1 deficient (*Fpr1*KO) mice in the presence or absence of Ent. In this experiment, we also included colon sections from myeloid differentiation factor 88 deficient (*Myd88*KO) mice, given that MyD88 is the downstream adaptor of all Toll-like receptors (except TLR3) that regulate most innate immune responses. Our results indicated WT colon explants were more responsive to Ent and upregulated secretion of keratinocyte-derived chemokine CXCL1 (KC, mouse ortholog of human IL-8) to a greater extent compared to *Fpr1*KO and *Myd88*KO colon explants (Figure 6(e)). These results suggest that the secretion of IL-8 in IECs in response to Ent may involve FPR1 and/or MyD88, *albeit* demonstration of this notion would require additional experimentation.

## Discussion

Iron is the second most abundant transition metal on the Earth’s crust, yet more than 2 billion people worldwide are afflicted with iron deficiency.^49^ To preserve iron sufficiency, our body expresses more than a dozen iron-binding and regulatory proteins to tightly control the economy of iron throughout its absorption, transport, storage, and recycling. Such acquisitiveness for iron displayed by host organisms has led various gut bacteria to produce iron-chelating siderophores as means to wage a ‘*tug-of-war*’ for this precious commodity.^29^ One such siderophore is Ent, the quintessential catecholate-type siderophore that exhibits the strongest, unparalleled affinity toward ferric iron (K_a_ = 10^49^ M^−1^).^36,37,40^ While Ent is classically viewed as an iron chelator, recent studies have begun to shed light on its other non-canonical functions such as promoting bacterial colonization, biofilm formation, and resistance to oxidative stress.^17-21^ We have previously reported that Ent can also interfere with the functions of neutrophils^16,23^ and macrophages,^15^ thus underscoring the importance of iron in immune responses. Intriguingly, a recent study by Qi et al. uncovered that the host also has the means to exploit bacterial Ent to facilitate cellular iron uptake and transport into the mitochondria.^50^ Such duality in the symbiotic/antagonistic relationship between gut commensals and their host in iron homeostasis is an area that warrants further studies, especially on how such interplay could impact health and disease.

Despite the potential advantage of Ent to strengthen physiological iron economy, excessive levels of iron in the mitochondria can also increase susceptibility toward oxidative damage. Accordingly, appropriate host defenses are required to fend off perturbation in iron homeostasis by bacterial siderophores during infection and/or gut dysbiosis. Epithelial cells lining the mucosal surfaces are the first cell-types to encounter invasive pathogens given their strategic location in our body. In response to inflammation, epithelial cells have been shown to upregulate the expression of Lcn2 to exert an important role in maintaining nutritional immunity against siderophore-expressing bacteria by binding and neutralizing a wide-range of carboxylate- and catecholate-type siderophores, such as Ent.^29,51^ Additionally IECs promote neutrophil migration to the site of infection or stress by secreting chemokines, such as IL-8.^52^ Pivotal studies by groups led by Bachman^28^ and Weisser^27^ and their colleagues have found that the A549 cell-line, a model for respiratory epithelial cells, can secrete IL-8 upon being treated with 50 μM of Ent. Their subsequent studies further affirm that such immune response to Ent is critical for the host defense against respiratory infection by *Kiebsiella pneumonia*.^53,54^ Since previous studies^27,28^ have only so far focused on respiratory epithelial cells which are rarely, if not at all, exposed to Ent under healthy conditions, we undertook this study to investigate whether IECs are sensitive or tolerant to Ent at concentrations employed in those prior studies. We address this notion herein by employing three different human IECs cell-lines, *i.e*. HT29, DLD-1 and Caco2/BBe cells, which we found to express varying levels of Lcn2. We demonstrated that iron-free, but not iron-bound, Ent can initiate an IL-8 response from human IECs in a dose-dependent manner.

Analogous to the report by Nelson et al.,^27^ we noted that only Ent was capable of inducing IL-8, which we did not observe with other known bacterial and fungal chelators. This prompts the critical question of what makes Ent particularly potent in inducing this pro-inflammatory chemokine. Nelson et al. observed that Ent could induce IL-8 and only when administered as iron-free^27^ and observed that exogenous Lcn2 could further increase the induction of IL-8 by promoting Ent internalization. In that study,^27^ the change in cellular iron was assessed indirectly by measuring the level of transferrin receptor 1 (TfR1), whose expression is known to be upregulated when labile cellular iron pools are depleted.^55^ Herein, we used the calcein-AM method^56^ to more directly determine and confirm that Ent is indeed capable of chelating the intracellular LIP in IECs. The degree of LIP chelation by Ent was highest in HT29 cells and lowest in Caco-2/BBe cells, which is inversely associated with their intra- and extra- cellular levels of Lcn2. This finding is consistent with the function of Lcn2 in neutralizing the various effects of Ent,^16,23,57^ thus high levels of Lcn2 in Caco-2/BBe cells could explain, in part, their lack of IL-8 response to Ent when compared to HT29 IECs. However, we do not rule out the possibility that Caco-2/BBe cells themselves were less responsive to Toll-like receptor (TLR) ligands in general. A prior study noted that, when challenged with bacterial flagellin (a TLR5 ligand), Caco-2/BBe cells secreted IL-8 *albeit* to a lesser degree when compared to HT29 cells.^58^ Notwithstanding this point, we noted a disparity in Lcn2 secretion between Caco-2/BBe and HT29 cells, which led us to test whether supplementing HT29 cells with exogenous recombinant Lcn2 could dampen Ent-induced IL-8 response. Our results assert that Lcn2 can indeed prevent Ent-induced IL-8 secretion, at least in the context of HT29 IECs. We also observed that Ent could also reduce or inhibit the expression of Lcn2 in DLD-1 and Caco-2/BBe cells, though the implication of this observation awaits further investigation. Likewise, possible mechanisms on how the role of Lcn2 in promoting/demoting Ent-induced IL-8 could differ between intestinal and respiratory epithelial cells also remains incompletely understood and requires further study.

Though the cellular iron-chelation activity of DFO was not measured in this study, we corroborated that DFO did not induce IL-8 in IECs. Presuming that Ent chelation of labile iron is superior to DFO in IECs, this would suggest that the iron-chelation activity of Ent is responsible, at least in part, for the induction of IL-8. This point is further supported by our observation that iron-bound Ent, which cannot chelate iron, could neither induce IL-8 or affect the intracellular iron pool in IECs. It is possible that the disparity between Ent and other siderophores could be due to its hydrophobic nature that facilitates the membrane permeability of Ent, whereas DFO is not cell-permeable due to its hydrophilicity.^59^ It is important to note that DFO, at a high concentration of 0.2 mM, has been reported to trigger the production of IL-8 in human IECs by activating the ERK1/2 and p38 kinase pathways.^41,42^ In line with this notion, we observed that 2,2 dipyridyl, despite being a highly cell-permeable intracellular iron chelator, could not induce IL-8 secretion from HT29 at concentrations less than 100 µM. These findings may suggest that having both a high affinity for iron and membrane-permeability could explain, in part, why Ent (K_a_ = 10^49^ M^−1^), but not DFO (K_a_ = 10^31^ M^−1^) nor DIP (K_a_ = 10^28^ M^−1^),^36-40^ could induce IL-8 responses in IECs at a lower dose of 25 μM.

Besides the superior kinetics of Ent, we envisaged that Ent mediated IL-8 secretion might be dependent on N-formyl peptide receptors (FPRs). FPRs are broadly expressed pattern recognition receptors, which can bind and induce responses to bacteria-derived peptides and amino acid derivatives.^43,60^ We considered the possibility that Ent could interact with FPRs given that the cyclic structure of Ent contains a tri-ester lactone of 2,3-dihydroxybenzoylserine, which is formed by an amide linkage of three 2,3-dihydroxybenzoic acid groups to three L-serine units.^47^ Our results indicate that Ent mediated IL-8 secretion may be FPR dependent as this response can be inhibited by Boc2, a widely used antagonist of FPRs, as well as by cyclosporine H, which is a selective inhibitor of FPR1. Though the possibility that Ent can activate FPR is worth considering, it is also plausible that inhibiting FPR via pharmacological inhibitors could induce a state of immune tolerance that dampens IECs IL-8 response to Ent. Aside from that, we also noted a lack of IL-8 response from MyD88 deficient colon cultures that were treated with Ent *ex vivo*. MyD88 is known to be a key adaptor protein that relays inflammatory signals and subsequent induction of cytokines including chemokines such as IL-8.^61^ We infer the possibility that the Ent-IL-8 axis may putatively signal through an inflammatory pathway upstream of MyD88. However, the exact role of FPR and MyD88 and how their pathways intersect within the Ent-IL-8 axis remains poorly understood and would certainly require further studies and validation.

In summary, our results demonstrated the role of Ent in promoting a pro-inflammatory response, chelating the intracellular LIP, and reducing basal ROS generation in IECs. The fact that Ent can induce an inflammatory response, as a single stimulus, from IECs may have clinical implications, especially when considered in the context of inflammatory bowel diseases (IBD). This notion coincides with the fact that *Enterobacteriaceae* bloom is one of the most consistent taxanomical shifts in the gut during IBD^62^ and that many members in this bacterial family (*e.g. E. coli*) produce Ent.^63^ Ent released in response to the iron-limiting condition known as hypoferremia of inflammation during IBD could exacerbate the disease not only by sustaining the influx of immune cells into the inflamed gut via IL-8, but also deplete the iron pool, which is needed for mucosal restitution. The inhibition of ROS production in IECs by Ent could additionally interfere with cellular processes downstream of ROS-dependent signaling, such as IECs proliferation^44^ and wound healing.^43^ Taken together, the findings from this study could aid in advancing our understanding on how perturbation of iron homeostasis in epithelial cells can affect innate immune and inflammatory responses.

## Materials and methods

### Reagents

Enterobactin (Ent; from *Escherichia coli*) procured from Sigma-Aldrich (St. Louis, MO) is supplied free of iron and endotoxin (≥98% purity; HPLC). Iron-free Pyoverdine (from *Pseudomonas fluorescens*), ferrichrome (from *Ustilago sphaerogena*), Deferiprone, deferoxamine mesylate salt (DFO; from *Streptomyces pilosus*), 2,3 dihydroxybenzoic acid (2,3-DHBA), lipopolysaccharide (LPS) from *E. coli* 0128:B12, dimethyl sulfoxide (DMSO), ferric chloride (FeCl3, Fe^3+^), 2,2ʹ-dipyridyl, and cyclosporin H were purchased from Sigma-Aldrich (St. Louis, MO). Flagellin (FliC) from *Salmonella enterica* subsp. *enterica serovar* Typhimurium (SL3201, ﬂjB-), a kind gift from Dr. Andrew Gewirtz, Georgia State University, was puriﬁed through sequential cation and anion-exchange chromatography as previously described.^52^ FITC Annexin-V apoptosis detection kit was purchased from Molecular Probes (Life Technologies, Columbus, OH). Chrome azurol S (CAS) was purchased from Acros Organics (Geel, Belgium). Recombinant mouse Lcn2 (*alias* neutrophil gelatinase-associated lipocalin) (rec-Lcn2; Cat# CM17) produced by a mammalian (human) expression system was procured from Novoprotein Scientific Inc. (Fremont, CA). Carrier-free rec-Lcn2 was supplied at a purity ≥95% as determined by reducing SDS-PAGE and free from endotoxin, siderophore, and iron.

### Mice

C57BL/6 J wild-type (WT) and myeloid differentiation primary response gene 88 global knockout (*Myd88*KO) mice were obtained from the Jackson Laboratory (Bar Harbor, ME) and bred in the animal vivarium at the University of Toledo. The offspring were cross-bred to generate their respective *Myd88*KO and WT littermates. Formyl peptide receptor global knockout (*Fpr1*KO)^64^ and their corresponding WT littermates on C57BL/6 J genetic background were from the colony of Dr. Camilla F. Wenceslau from the University of Toledo. All mice used in the present study were bred and maintained under specific pathogen-free conditions at 23°C with a 12-h light/dark phase cycle. Mice were housed in cages containing corn cob bedding (Bed-O-Cob, The Andersons Co.) and nestlets (Cat # CABFM00088, Ancare), and fed *ad libitum* (LabDiet 5020 for breeders and LabDiet 5001 for weaned mice). All the animal experiments were approved by The Institutional Animal Care and Use Committee (IACUC) at the University of Toledo.

### Human model intestinal epithelial cells

The human colonic epithelial cell line HT29 (kindly gifted by Dr. Terry Hinds, University of Toledo) was maintained in McCoy’s 5A medium (Hyclone) supplemented with 1.5 mM L-glutamine and 2.2 g/L sodium bicarbonate, 10% (v/v) heat-inactivated fetal bovine serum (FBS), 100 IU/ml penicillin, and 100 µg/ml streptomycin at 37°C in a humidified incubator with 5% CO_2_. Culture media was changed every 2 days, and the cell-line was trypsinized with 0.25% trypsin-EDTA solution (Sigma) following standard procedures.

The human colonic epithelial cell-line DLD-1 (kindly gifted by Dr. Sivarajan Kumarasamy, University of Toledo) was sub-cultured and maintained in RPMI-1640 Medium containing 10% (v/v) FBS, 100 IU/ml penicillin, and 100 µg/ml streptomycin at 37°C in a humidified 5% CO_2_ incubator. Culture media was changed 2 to 3 times per week, and the cell-line was trypsinized with 0.25% trypsin-EDTA solution as per standard procedures.

The human colonic enterocyte cell-line Caco-2/BBe (kindly gifted by Dr. William Scott Crawley, University of Toledo) was maintained in Dulbecco’s Modified Eagle’s Medium (DMEM) supplemented with 20% (v/v) FBS, 100 IU/ml penicillin, and 100 µg/ml streptomycin at 37°C in a humidified 5% CO_2_ incubator. Culture media was replaced 2 times per week, and the cells were trypsinized with 0.25% trypsin-EDTA solution as per standard procedures.

### Cell stimulation with enterobactin

At 90–95% confluency, HT29 (8–10 passages), DLD-1 (10–15 passages), and Caco-2/BBe (10–12 passages) cells were seeded in 24 well plates (1.0 x 10^6^ cells/ml) and challenged with either Ent (25 μM), FeCl_3_ (Fe^3+^, 25 μM) or Ent+FeCl_3_ (1:1 ratio) prepared in DMSO (vehicle) in serum-free media supplemented with 1% penicillin-streptomycin. After 24 h, culture supernatants were analyzed for IL-8 and Lcn2 production via ELISA. In a separate experiment, HT29 cells were seeded in 12 well plates (2.0x10^6^ cells/ml) or 24 well plates (1.0 x 10^6^ cells/ml), pre-treated with FliC (100 ng/ml), LPS (1 µg/ml) or IFN-γ (100 IU), and then challenged with either Ent (25 μM), FeCl_3_ (Fe^3+^, 25 μM) or Ent+FeCl_3_ (1:1 ratio) in serum-free media supplemented with 1% penicillin-streptomycin. After 24 h, culture supernatants were analyzed for IL-8 secretion via ELISA.

In a separate experiment, HT29 cells were seeded in 24 well plates (1.0 x 10^6^ cells/ml) in serum-free media supplemented with 1% penicillin-streptomycin. Cells received the following treatment: (i) vehicle control, (ii) only rec-Lcn2 (15 µg/ml; 0.625 μM), (iii) only Ent (15 µg/ml; 25 μM), (iv) pre-treated with Ent for 1 h and then rec-Lcn2, (v) pre-treated with Ent for 1 h and then rec-Lcn2, (vi) rec-Lcn2 and Ent pre-incubated for 10 min at room temperature to allow complex formation before adding to cells. After 24 h, culture supernatants were examined for IL-8 secretion via ELISA. The rationale for using mouse recombinant Lcn2 was based on the study by Nelson et al.^27^ and other studies, which demonstrate that both mouse and human Lcn2 have comparable properties.^57,65,66^ Though the overall similarity in their amino acid sequence is 62%, both human and mouse Lcn2 share a conserved sequence of amino acids that participates in siderophore binding and in their uptake into mammalian cells.^57^

For inhibition assay with FPR antagonists, HT29 cell monolayers were pre-incubated with either Boc2 (0–50 µM, 1 h) or cyclosporin H (CspH, 0–10 µM) for 1 h and then treated with Ent (25 µM) for 24 h in serum-free media supplemented with 1% penicillin-streptomycin. Culture supernatants were assayed for IL-8 secretion via ELISA. In another experiment, HT29 cell monolayers were stimulated overnight with 25 µM of either Ent, DFO, deferiprone, ferrichrome, pyoverdine, DHBA, or 2,2ʹ-dipyridyl (0–100 µM) for 24 h in serum-free media supplemented with 1% penicillin-streptomycin. DMSO was used as vehicle control. Culture supernatants were quantified for IL-8 production via ELISA.

### Chrome Azurol S (CAS) assay

CAS agar plates and liquid reagents were prepared as previously described by Schwyn and Neilands.^30,67^ The principle of the assay is that CAS remains blue when complexed with iron, but changes to orange when the iron is chelated by siderophores. Equal concentrations of Ent (0.25–5 mM) were incubated on a CAS agar plate and monitored over 0–12 h for the formation of orange halo. To quantify iron chelation, Ent (0–50 µM) or Ent+ Fe^3+^ (0–50 µM) were added to the CAS liquid reagent (100 μl), incubated for 20 min at room temperature, and absorbance was measured at 630 nm. Percent iron chelation was calculated using 0–100 µM of pyrocatechol as a positive control as described previously.^30^

### Lactate dehydrogenase assay

Lactate dehydrogenase (LDH) levels in culture supernatants were measured using a kit from Randox (Crumlin, UK) according to the manufacturer’s instructions.

### Intracellular reactive oxygen species measurement

HT29 monolayers were treated with Ent (25 µM), Fe^3+^ (25 µM) or Ent+Fe^3+^ at equimolar ratio for 24 h. Cells were washed with PBS, stained with 5 µM CellROX® Deep Red dye (Molecular probes) for 30 min at 37°C in the dark, and then washed twice with PBS. Fluorescence was measured by Accuri c6 flow cytometer (BD Biosciences, San Jose, CA) and analyzed using the Accuri C6 software (BD Biosciences, San Jose, CA). Intracellular ROS was expressed as mean fluorescence intensity (MFI).

### Intracellular labile iron measurement

HT29, DLD-1, and Caco-2/BBe cells were incubated for 15 min at 37°C and 5% CO_2_ with 0.5 μM calcein acetoxymethyl ester (Sigma) to allow for formation of calcein-LIP complex in the cytosol. The cells were washed twice and treated with either Ent (25 µM) or Ent+ Fe^3+^ (at equimolar ratio) for 3 h. In principle, the iron-chelating property of Ent would compete for the LIP, thus releasing calcein whose fluorescence can be quantified based on the change in mean fluorescence intensity compared with control (ΔF).^32,33^ Following washing with PBS, cells were analyzed by using Accuri c6 flow cytometer (BD Biosciences, San Jose, CA): cells were gated on forward/side scatter plot and presented as cell count against calcein positivity detected on FL1 channel. The MFI was determined using the Accuri c6 Software (BD Biosciences, San Jose, CA). The magnitude of iron chelation (LIP, ΔF) was calculated by subtracting the difference in the MFI, before and after treatment, with Ent as previously described.^16,68,69^

### Apoptosis assay

HT29 cells (2.0x10^6^ cells/well) prepared in 1 ml incomplete McCoy’s 5A media were plated in 12 well plates. Ent (25 μM) was added to their respective wells and incubated for 0–24 h at 37ºC and 5% CO_2_. Cell viability and apoptosis were measured using the FITC Annexin-V apoptosis detection kit (BD Biosciences) according to manufacturer’s instruction. Results were acquired via flow cytometry (Accuri C6, BD Biosciences) and analyzed by FlowJo software (Becton Dickinson). Results were presented as the percentage of early and late apoptotic (Annexin-V single-positive and Annexin-V+ propidium iodide double-positive, PI) cells.

### Organ ex vivo culture

Two centimeter sections of the colon (below the cecum) from WT, *Fpr1*KO and *MyD88*KO mice were collected and cultured in serum-free DMEM media (Sigma) supplemented with 1% penicillin-streptomycin (Sigma). After two washes with sterile PBS (37°C), colon sections were transferred to 12 well culture plates (Corning) containing 1 mL of serum-free DMEM media with 1% penicillin-streptomycin and incubated for 24 h at 37°C in CO_2_ incubator with and without Ent (25 µM). The cultures were then centrifuged (10,000 g; 4°C) and clear supernatant was collected for measuring keratinocyte-derived chemokine CXCL1 (KC; a murine homolog of human IL-8) via ELISA.

### ELISA

Human IL-8 and lipocalin 2 (Lcn2) and mouse keratinocyte-derived chemokine CXCL1 (KC) were quantified in culture supernatant and cell lysates using Duoset ELISA kits procured from R&D Systems (Minneapolis, MN) according to manufacturer’s instructions.

### Statistical analysis

All *in vitro* experiments were performed in triplicates and data were presented as representative of three independent experiments. Results were expressed as mean ± SEM. Statistical significance between two groups was analyzed using unpaired, two-tailed *t-test*. Data from more than two groups were compared using one-way analysis of variance (ANOVA) followed by Dunnett’s *post hoc* test (to compare the mean of each column with the mean control column) or Tukey’s multiple comparison tests (to compare the mean of each column with the mean of every other column). All statistical analyses were performed with the GraphPad Prism 7.0 software (GraphPad Inc, La Jolla, CA). *p* < .05 was considered as statistically significant and denoted as * *p* < .05, ** *p* < .01, *** *p* < .001 and **** *p* < .0001.

## Abbreviations

Ent: enterobactin,
LIP: labile iron pool
MFI: mean fluorescence intensity
WT: wild-Type
DFO: deferoxamine mesylate
IECs: intestinal epithelial cells
Lcn2: lipocalin 2
FPR: formyl peptide receptor
IL-8: interleukin 8
CAS: chrome azurol S
LPS: lipopolysaccharide
LDH: lactate dehydrogenase
DHBA: 2,3 dihydroxybenzoic acid
DIP: 2,2 dipyridyl

## References

[1] Groschwitz KR, Hogan SP. Intestinal barrier function: molecular regulation and disease pathogenesis. J Allergy Clin Immunol. 2009;124:3–20;quiz 1–2. doi:10.1016/j.jaci.2009.05.038. 19560575 PMC4266989

[2] Okumura R, Takeda K. Roles of intestinal epithelial cells in the maintenance of gut homeostasis. Exp Mol Med. 2017;49:e338. doi:10.1038/emm.2017.20. 28546564 PMC5454438

[3] Yu Y, Zeng H, Lyons S, Carlson A, Merlin D, Neish AS, Gewirtz AT. TLR5-mediated activation of p38 MAPK regulates epithelial IL-8 expression via posttranscriptional mechanism. Am J Physiol Gastrointest Liver Physiol. 2003;285(2):G282–90. doi:10.1152/ajpgi.00503.2002. 12702497

[4] Gewirtz AT, Navas TA, Lyons S, Godowski PJ, Madara JL. Cutting edge: bacterial flagellin activates basolaterally expressed TLR5 to induce epithelial proinflammatory gene expression. J Immunol. 2001;167:1882–1885. doi:10.4049/jimmunol.167.4.1882. 11489966

[5] Fasano A. Zonulin and its regulation of intestinal barrier function: the biological door to inflammation, autoimmunity, and cancer. Physiol Rev. 2011;91:151–175. 21248165 10.1152/physrev.00003.2008

[6] Maloy KJ, Powrie F. Intestinal homeostasis and its breakdown in inflammatory bowel disease. Nature. 2011;474:298–306. doi:10.1038/nature10208. 21677746

[7] Cani PD, Amar J, Iglesias MA, Poggi M, Knauf C, Bastelica D, Neyrinck AM, Fava F, Tuohy KM, Chabo C. Metabolic endotoxemia initiates obesity and insulin resistance. Diabetes. 2007;56(7):1761–1772. doi:10.2337/db06-1491. 17456850

[8] Liang S, Guo XK, Ou J, Huang R, Xue Q, Zhang B, Yeonseok Chung Y, Wu W, Dong C, Yang X, Hu X. Nutrient sensing by the intestinal epithelium orchestrates mucosal antimicrobial defense via translational control of Hes1. Cell Host Microbe. 2019;25:706–18e7. 31053533 10.1016/j.chom.2019.03.012

[9] Borregaard N, Cowland JB. Neutrophil gelatinase-associated lipocalin, a siderophore-binding eukaryotic protein. Biometals. 2006;19(2):211–215. doi:10.1007/s10534-005-3251-7. 16718606

[10] Ratledge C, Dover LG. Iron metabolism in pathogenic bacteria. Annu Rev Microbiol. 2000;54:881–941. doi:10.1146/annurev.micro.54.1.881. 11018148

[11] Abergel RJ, Warner JA, Shuh DK, Raymond KN. Enterobactin protonation and iron release: structural characterization of the salicylate coordination shift in ferric enterobactin. J Am Chem Soc. 2006;128:8920–8931. doi:10.1021/ja062046j. 16819888 PMC3188320

[12] Fischbach MA, Lin H, Liu DR, Walsh CT. How pathogenic bacteria evade mammalian sabotage in the battle for iron. Nat Chem Biol. 2006;2:132–138. doi:10.1038/nchembio771. 16485005

[13] Luo M, Lin H, Fischbach MA, Liu DR, Walsh CT, Groves JT. Enzymatic tailoring of enterobactin alters membrane partitioning and iron acquisition. ACS Chem Biol. 2006;1:29–32. doi:10.1021/cb0500034. 17163637

[14] Saha P, Yeoh BS, Xiao X, Golonka RM, Kumarasamy S, Vijay-Kumar M. Enterobactin, an iron chelating bacterial siderophore, arrests cancer cell proliferation. Biochem Pharmacol. 2019;168:71–81. doi:10.1016/j.bcp.2019.06.017. 31228465 PMC6733644

[15] Saha P, Xiao X, Yeoh BS, Chen Q, Katkere B, Kirimanjeswara GS, Vijay-Kumar M. The bacterial siderophore enterobactin confers survival advantage to Salmonella in macrophages. Gut Microbes. 2019;10:412–423. doi:10.1080/19490976.2018.1546519. 30449241 PMC6546333

[16] Saha P, Yeoh BS, Olvera RA, Xiao X, Singh V, Awasthi D, Subramanian BC, Chen Q, Dikshit M, Wang Y. Bacterial siderophores hijack neutrophil functions. The Journal of Immunology. 2017;198(11):4293–4303. doi:10.4049/jimmunol.1700261. 28432145 PMC5470626

[17] Pi H, Jones SA, Mercer LE, Meador JP, Caughron JE, Jordan L, Newton SM, Conway T, Klebba PE. Role of catecholate siderophores in gram-negative bacterial colonization of the mouse gut. PLoS One. 2012;7(11):e50020. doi:10.1371/journal.pone.0050020. 23209633 PMC3510177

[18] May T, Okabe S. Enterobactin is required for biofilm development in reduced-genome Escherichia coli. Environ Microbiol. 2011;13(12):3149–3162. doi:10.1111/j.1462-2920.2011.02607.x. 21980953

[19] Peralta DR, Adler C, Corbalan NS, Paz Garcia EC, Pomares MF, Vincent PA. Enterobactin as part of the oxidative stress response repertoire. PloS One. 2016;11:e0157799. doi:10.1371/journal.pone.0157799. 27310257 PMC4911079

[20] Adler C, Corbalan NS, Peralta DR, Pomares MF, de Cristobal RE, Vincent PA. The alternative role of enterobactin as an oxidative stress protector allows Escherichia coli colony development. PloS One. 2014;9:e84734. doi:10.1371/journal.pone.0084734. 24392154 PMC3879343

[21] Achard ME, Chen KW, Sweet MJ, Watts RE, Schroder K, Schembri MA, McEwan A. An antioxidant role for catecholate siderophores in Salmonella. Biochem J. 2013;454:543–549. doi:10.1042/BJ20121771. 23805839

[22] Coker MS, Forbes LV, Plowman-Holmes M, Murdoch DR, Winterbourn CC, Kettle AJ. Interactions of staphyloxanthin and enterobactin with myeloperoxidase and reactive chlorine species. Arch Biochem Biophys. 2018;646:80–89. doi:10.1016/j.abb.2018.03.039. 29614256

[23] Singh V, Yeoh BS, Xiao X, Kumar M, Bachman M, Borregaard N, Joe B, Vijay-Kumar M. Interplay between enterobactin, myeloperoxidase and lipocalin 2 regulates E. coli survival in the inflamed gut. Nat Commun. 2015;6(1):7113. doi:10.1038/ncomms8113. 25964185 PMC6336494

[24] Valdebenito M, Muller SI, Hantke K. Special conditions allow binding of the siderophore salmochelin to siderocalin (NGAL-lipocalin). FEMS Microbiol Lett. 2007;277:182–187. doi:10.1111/j.1574-6968.2007.00956.x. 18031338

[25] Coorens M, Rao A, Grafe SK, Unelius D, Lindforss U, Agerberth B, Mjösberg J, Bergman P. Innate lymphoid cell type 3-derived interleukin-22 boosts lipocalin-2 production in intestinal epithelial cells via synergy between STAT3 and NF-kappaB. J Biol Chem. 2019;294:6027–6041. doi:10.1074/jbc.RA118.007290. 30782844 PMC6463718

[26] Wu H, Santoni-Rugiu E, Ralfkiaer E, Porse BT, Moser C, Hoiby N, Borregaard N, Cowland JB. Lipocalin 2 is protective against E. coli pneumonia. Respir Res. 2010;11(1):96. doi:10.1186/1465-9921-11-96. 20633248 PMC2912245

[27] Nelson AL, Ratner AJ, Barasch J, Weiser JN. Interleukin-8 secretion in response to aferric enterobactin is potentiated by siderocalin. Infect Immun. 2007;75:3160–3168. doi:10.1128/IAI.01719-06. 17420239 PMC1932857

[28] Holden VI, Lenio S, Kuick R, Ramakrishnan SK, Shah YM, Bachman MA. Bacterial siderophores that evade or overwhelm lipocalin 2 induce hypoxia inducible factor 1alpha and proinflammatory cytokine secretion in cultured respiratory epithelial cells. Infect Immun. 2014;82:3826–3836. doi:10.1128/IAI.01849-14. 24980968 PMC4187820

[29] Golonka R, Yeoh BS, Vijay-Kumar M. The Iron Tug-of-War between Bacterial Siderophores and Innate Immunity. J Innate Immun. 2019;11:249–262. 30605903 10.1159/000494627 PMC6487204

[30] Xiao X, Yeoh BS, Saha P, Tian Y, Singh V, Patterson AD, Vijay-Kumar M. Modulation of urinary siderophores by the diet, gut microbiota and inflammation in mice. J Nutr Biochem. 2017;41:25–33. 27951517 10.1016/j.jnutbio.2016.11.014 PMC5315603

[31] Kakhlon O, Cabantchik ZI. The labile iron pool: characterization, measurement, and participation in cellular processes(1). Free Radic Biol Med. 2002;33:1037–1046. doi:10.1016/S0891-5849(02)01006-7. 12374615

[32] Cabantchik ZI, Glickstein H, Milgram P, Breuer W. A fluorescence assay for assessing chelation of intracellular iron in a membrane model system and in mammalian cells. Anal Biochem. 1996;233:221–227. doi:10.1006/abio.1996.0032. 8789722

[33] Glickstein H, El RB, Shvartsman M, Cabantchik ZI. Intracellular labile iron pools as direct targets of iron chelators: a fluorescence study of chelator action in living cells. Blood. 2005;106:3242–3250. doi:10.1182/blood-2005-02-0460. 16020512

[34] Chan FK, Moriwaki K, De Rosa MJ. Detection of necrosis by release of lactate dehydrogenase activity. Methods Mole Biol. 2013;979:65–70. 10.1007/978-1-62703-290-2_7 PMC3763497 23397389

[35] Stark GR, Kerr IM, Williams BR, Silverman RH, Schreiber RD. How cells respond to interferons. Annu Rev Biochem. 1998;67:227–264. doi:10.1146/annurev.biochem.67.1.227. 9759489

[36] Miethke M, Marahiel MA. Siderophore-based iron acquisition and pathogen control. Microbiol Mol Biol Rev. 2007;71:413–451. 17804665 10.1128/MMBR.00012-07 PMC2168645

[37] Raymond KN, Dertz EA, Kim SS. Enterobactin: an archetype for microbial iron transport. Proc Natl Acad Sci U S A. 2003;100:3584–3588. 12655062 10.1073/pnas.0630018100 PMC152965

[38] Richardson DR, Hefter GT, May PM, Webb J, Baker E. Iron chelators of the pyridoxal isonicotinoyl hydrazone class. III. Formation constants with calcium(II), magnesium(II) and zinc(II). Biol Met. 1989;2:161–167. doi:10.1007/BF01142555. 2490071

[39] Goodwin JF, Whitten CF. Chelation of ferrous sulphate solutions by desferrioxamine B. Nature. 1965;205:281–283. doi:10.1038/205281b0. 14270711

[40] Loomis LD, Raymond KN. Solution equilibria of enterobactin and metal-enterobactin complexes. Inorg Chem. 1991;30:906–911. doi:10.1021/ic00005a008.

[41] Choi EY, Park ZY, Choi EJ, Oh HM, Lee S, Choi SC, Lee KM, Im SH, Chun JS, Jun CD. Transcriptional regulation of IL-8 by iron chelator in human epithelial cells is independent from NF-kappaB but involves ERK1/2- and p38 kinase-dependent activation of AP-1. J Cell Biochem. 2007;102:1442–1457. 17471497 10.1002/jcb.21367

[42] Choi EY, Kim EC, Oh HM, Kim S, Lee HJ, Cho EY, Yoon KH, Kim EA, Han WC, Choi SC, et al. Iron chelator triggers inflammatory signals in human intestinal epithelial cells: involvement of p38 and extracellular signal-regulated kinase signaling pathways. J Immunol. 2004;172:7069–7077. 15153529 10.4049/jimmunol.172.11.7069

[43] Alam A, Leoni G, Wentworth CC, Kwal JM, Wu H, Ardita CS, Swanson PA, Lambeth JD, Jones RM, Nusrat A. Redox signaling regulates commensal-mediated mucosal homeostasis and restitution and requires formyl peptide receptor 1. Mucosal Immunol. 2014;7(3):645–655. doi:10.1038/mi.2013.84. 24192910 PMC3999246

[44] Moll F, Walter M, Rezende F, Helfinger V, Vasconez E, De Oliveira T, Greten FR, Olesch C, Weigert A, Radeke HH. NoxO1 Controls Proliferation of Colon Epithelial Cells. Front Immunol. 2018;9:973. doi:10.3389/fimmu.2018.00973. 29867954 PMC5951971

[45] Nakamura T, Naguro I, Ichijo H. Iron homeostasis and iron-regulated ROS in cell death, senescence and human diseases. Biochim Biophys Acta Gen Subj. 2019;1863(9):1398–1409. doi:10.1016/j.bbagen.2019.06.010. 31229492

[46] Babbin BA, Jesaitis AJ, Ivanov AI, Kelly D, Laukoetter M, Nava P, Parkos CA, Nusrat A. Formyl peptide receptor-1 activation enhances intestinal epithelial cell restitution through phosphatidylinositol 3-kinase-dependent activation of Rac1 and Cdc42. J Immunol. 2007;179(12):8112–8121. doi:10.4049/jimmunol.179.12.8112. 18056353

[47] Leslie AD, Daneshfar R, Volmer DA. Infrared multiphoton dissociation of the siderophore enterobactin and its Fe(III) complex. Influence of Fe(III) binding on dissociation kinetics and relative energetics. J Am Soc Mass Spectrom. 2007;18(4):632–641. doi:10.1016/j.jasms.2006.11.011. 17208008

[48] Nawaz IM, Chiodelli P, Rezzola S, Paganini G, Corsini M, Lodola A, Di Ianni A, Mor M, Presta M. N-tert-butyloxycarbonyl-Phe-Leu-Phe-Leu-Phe (BOC2) inhibits the angiogenic activity of heparin-binding growth factors. Angiogenesis. 2018;21(1):47–59. doi:10.1007/s10456-017-9581-6. 29030736

[49] McLean E, Cogswell M, Egli I, Wojdyla D, de Benoist B. Worldwide prevalence of anaemia, WHO Vitamin and mineral nutrition information system, 1993-2005. Public Health Nutr. 2009;12:444–454. 18498676 10.1017/S1368980008002401

[50] Qi B, Han M. Microbial siderophore enterobactin promotes mitochondrial iron uptake and development of the host via interaction with ATP synthase. Cell. 2018;175(2):571–82e11. doi:10.1016/j.cell.2018.07.032. 30146159

[51] Xiao X, Yeoh BS, Vijay-Kumar M. Lipocalin 2: an emerging player in iron homeostasis and inflammation. Annu Rev Nutr. 2017;37(1):103–130. doi:10.1146/annurev-nutr-071816-064559. 28628361

[52] Gewirtz AT, Simon PO Jr., Schmitt CK, Taylor LJ, Hagedorn CH, O’Brien AD, Neish AS, Madara JL. Salmonella typhimurium translocates flagellin across intestinal epithelia, inducing a proinflammatory response. J Clin Invest. 2001;107:99–109. doi:10.1172/JCI10501. 11134185 PMC198545

[53] Holden VI, Breen P, Houle S, Dozois CM, Bachman MA. Klebsiella pneumoniae siderophores induce inflammation, bacterial dissemination, and HIF-1alpha stabilization during pneumonia. mBio. 2016;7. doi:10.1128/mBio.01397-16. PMC5021805 27624128

[54] Bachman MA, Miller VL, Weiser JN. Mucosal lipocalin 2 has pro-inflammatory and iron-sequestering effects in response to bacterial enterobactin. PLoS Pathog. 2009;5:e1000622. doi:10.1371/journal.ppat.1000622. 19834550 PMC2757716

[55] Casey JL, Hentze MW, Koeller DM, Caughman SW, Rouault TA, Klausner RD, Harford J. Iron-responsive elements: regulatory RNA sequences that control mRNA levels and translation. Science. 1988;240:924–928. doi:10.1126/science.2452485. 2452485

[56] Tenopoulou M, Kurz T, Doulias PT, Galaris D, Brunk UT. Does the calcein-AM method assay the total cellular ‘labile iron pool’ or only a fraction of it? Biochem J. 2007;403:261–266. doi:10.1042/BJ20061840. 17233627 PMC1874234

[57] Goetz DH, Holmes MA, Borregaard N, Bluhm ME, Raymond KN, Strong RK. The neutrophil lipocalin NGAL is a bacteriostatic agent that interferes with siderophore-mediated iron acquisition. Mol Cell. 2002;10:1033–1043. doi:10.1016/S1097-2765(02)00708-6. 12453412

[58] Sun J, Fegan PE, Desai AS, Madara JL, Hobert ME. Flagellin-induced tolerance of the Toll-like receptor 5 signaling pathway in polarized intestinal epithelial cells. Am J Physiol Gastrointest Liver Physiol. 2007;292:G767–78. doi:10.1152/ajpgi.00447.2006. 17138965

[59] Alta RY, Vitorino HA, Goswami D, Liria CW, Wisnovsky SP, Kelley SO, Machini MT, Espósito BP. Mitochondria-penetrating peptides conjugated to desferrioxamine as chelators for mitochondrial labile iron. PLoS One. 2017;12:e0171729. doi:10.1371/journal.pone.0171729. 28178347 PMC5298241

[60] Chen K, Liu M, Liu Y, Yoshimura T, Shen W, Le Y, Durum S, Gong W, Wang C, Gao J-L. Formylpeptide receptor-2 contributes to colonic epithelial homeostasis, inflammation, and tumorigenesis. J Clin Invest. 2013;123(4):1694–1704. doi:10.1172/JCI65569. 23454745 PMC3613917

[61] Warner N, Nunez G. MyD88: a critical adaptor protein in innate immunity signal transduction. J Immunol. 2013;190(1):3–4. doi:10.4049/jimmunol.1203103. 23264668

[62] Kostic AD, Xavier RJ, Gevers D. The microbiome in inflammatory bowel disease: current status and the future ahead. Gastroenterology. 2014;146:1489–1499. doi:10.1053/j.gastro.2014.02.009. 24560869 PMC4034132

[63] Payne SM. Iron and virulence in the family Enterobacteriaceae. Crit Rev Microbiol. 1988;16:81–111. doi:10.3109/10408418809104468. 3067977

[64] Wenceslau CF, McCarthy CG, Szasz T, Calmasini FB, Mamenko M, Webb RC. Formyl peptide receptor-1 activation exerts a critical role for the dynamic plasticity of arteries via actin polymerization. Pharmacol Res. 2019;141:276–290. doi:10.1016/j.phrs.2019.01.015. 30639374 PMC6391177

[65] Flo TH, Smith KD, Sato S, Rodriguez DJ, Holmes MA, Strong RK, Akira S, Aderem A. Lipocalin 2 mediates an innate immune response to bacterial infection by sequestrating iron. Nature. 2004;432(7019):917–921. doi:10.1038/nature03104. 15531878

[66] Kjeldsen L, Cowland JB, Borregaard N. Human neutrophil gelatinase-associated lipocalin and homologous proteins in rat and mouse. Biochim Biophys Acta. 2000;1482:272–283. 11058768 10.1016/s0167-4838(00)00152-7

[67] Schwyn B, Neilands JB. Universal chemical assay for the detection and determination of siderophores. Anal Biochem. 1987;160:47–56. doi:10.1016/0003-2697(87)90612-9. 2952030

[68] Saha P, Xiao X, Yeoh BS, Chen Q, Katkere B, Kirimanjeswara GS, et alVijay-Kumar M. The bacterial siderophore enterobactin confers survival advantage to Salmonella in macrophages. Gut Microbes. 2019;10(3):412–423 30449241 10.1080/19490976.2018.1546519 PMC6546333

[69] Prus E, Fibach E. Flow cytometry measurement of the labile iron pool in human hematopoietic cells. Cytometry A. 2008;73:22–27. doi:10.1002/cyto.a.20491. 18044720

